# Gender Disparities in Lipid Goal Attainment among Type 2 Diabetes Outpatients with Coronary Heart Disease: Results from the CCMR-3B Study

**DOI:** 10.1038/s41598-017-13066-z

**Published:** 2017-10-04

**Authors:** Xiaomei Zhang, Linong Ji, Xingwu Ran, Benli Su, Qiuhe Ji, Dayi Hu

**Affiliations:** 1 Peking University International Hospital, Department of Endocrinology and Metabolism, Beijing, 102206 China; 2 Peking University People’s Hospital, Department of Endocrinology and Metabolism, Beijing, 100044 China; 3 West China Hospital, Sichuan University, Department of Endocrinology and Metabolism, Chengdu, 610041 China; 4 The Second Affiliated Hospital of Dalian Medical University, Department of Endocrinology, Dalian, 116027 China; 5 Xijing Hospital, Forth Military Medical University, Department of Endocrinology, Xi’an, 710032 China; 6 Peking University People’s Hospital, Department of Cardiology, Beijing, 100044 China

## Abstract

Our study was aimed to investigate the gender disparities in lipid goal attainment among type 2 diabetes outpatients with concomitant coronary heart disease (CHD) and explore potential risk factors. We performed the present analysis using data from a nationally representative epidemiologic study. The therapeutic goal was defined as achieving a low-density lipoprotein cholesterol (LDL-C) <1.8 mmol/L. A total of 1721 male and 2072 female type 2 diabetes outpatients with established CHD were identified. Compared with men, women had higher levels of total cholesterol (4.98 vs. 4.46 mmol/L; p < 0.001), LDL-C (2.82 vs. 2.54 mmol/L; p < 0.001), and triglycerides (2.02 vs. 1.79 mmol/L; p < 0.001), but not hemoglobin A1c (7.47% vs. 7.50%; p = 0.597). The proportion of women received lipid-lowering therapy was lower (38.1% vs. 48.2%; p < 0.001). The percentages of patients who achieved the LDL-C goal were higher among men. Multivariable regression analysis indicated that the odds ratio for lipid goal attainment due to the gender difference was 0.61 after adjusting confounders. The inability to achieve LDL-C goals in women with type 2 diabetes and CHD is apparently greater than that in men. This finding underscores the importance of initiatives to establish a more aggressive lipid management strategy for women to overcome gender imbalances.

## Introduction

Diabetes mellitus is a common health concern worldwide. The global prevalence of type 2 diabetes is approximately 8.3% among adults, and an estimated 592 million individuals are predicted to be affected by 2035^1^. In China, the estimated prevalence of type 2 diabetes in adults is 11.6%^2^. Diabetes mellitus is a well-known risk factor for coronary heart disease (CHD) in both women and men^3^. However, women with diabetes have a more than 40% greater risk of incident CHD compared with men with diabetes^4^. CHD is the leading cause of death among diabetes patients^5^. Patients with type 2 diabetes have an increased incidence of cardiovascular risk factors. Among these risk factors, lipid abnormalities are an essential determinant of cardiovascular risk in type 2 diabetes^6^. Approximately 64% of diabetes patients also have high cholesterol levels^7^, and the coexistence of diabetes and dyslipidemia promotes atherosclerosis of the coronary arteries and thus increases the risk of CHD. Therefore, dyslipidemia management is an important strategy for the prevention of CHD in this high-risk population.

The control of dyslipidemia in patients with type 2 diabetes has posed a serious challenge. Diabetic dyslipidemia is usually characterized by a low serum level of high-density lipoprotein cholesterol (HDL-C) and high levels of triglycerides and low- density lipoprotein cholesterol (LDL-C)^8^. LDL-C reduction is a major target for CHD prevention in type 2 diabetes patients^9^. The American Diabetes Association (ADA) guidelines recommended that the LDL-C level should be <2.6 mmol/L in diabetes patients, with an optional goal of <1.8 mmol/L in those with both diabetes and CHD^10^. Appropriate lipid management can reduce cardiovascular complications in individuals with diabetes or prediabetes^11^. However, despite aggressive lipid-lowering management, a significant proportion of diabetes patients do not achieve lipid control goal, particularly among women^12^. Gender differences in lipid management may be a particularly important contributor to suboptimal cardiovascular outcomes in women with diabetes and CHD. Several studies have shown that compared with diabetic men, diabetic women with dyslipidemia are less likely to be treated with lipid-lowering agents or to achieve the optimal LDL-C goal^13–17^. Still, there is a paucity of data regarding national estimates of gender-based disparities in lipid goal attainment in China. Furthermore, little is known about potential factors contributing to this gender imbalance. Here we present the first study to investigate gender disparities in lipid goal attainment in Chinese patients with type 2 diabetes mellitus and CHD. We performed a post hoc analysis of the China Cardiometabolic Registries (CCMR)-3B study results to investigate the gender disparities in lipid goal attainment among Chinese outpatients who had type 2 diabetes mellitus concomitant with CHD as well as to explore the potential risk factors for gender differences.

## Results

### General patient characteristics

Table 1 displays the characteristics of the included patients, and Supplemental Table 1 shows the distribution of enrolled patients and their social-economic status. A total of 3793 outpatients (1721 men and 2072 women) with type 2 diabetes and concomitant with CHD were identified. The mean ages of men and women were 66.4 and 69.1 years, respectively. There were statistically significant differences in age, waist circumstance, smoking history, alcohol consumption history, hypertension, diabetes duration, and mean levels of SBP, DBP, LDL-C, TC, TG, and HDL-C (all p < 0.05) between the men and the women, but no differences in the mean HbA1c level (7.47% vs. 7.50%; p = 0.597) or BMI (25.1 kg/m^2^ vs 25.3 kg/m^2^; p = 0.095). Moreover, the percentages of patient ≥60 years of age and with hypertension were higher in women than in men, as were the mean values of LDL-C, TC, and TG.

**Table 1:** Baseline characteristics of the included patients.

	Men (N = 1721)	Women (N = 2072)	p
Age (years), mean ± SD (n)	66.4 ± 10.78 (1717)	69.1 ± 9.13 (2068)	<0.001
≥60 years, % (n/N)	72.1% (1240/1721)	82.6% (1708/2068)	<0.001
Waist circumstance	88.0 ± 7.77 (1485)	79.4 ± 6.01 (1199)	<0.001
BMI (kg/m^2^), mean ± SD (n)	25.1 ± 3.27 (1721)	25.3 ± 3.97 (2072)	0.095
≥24 kg/m^2^, % (n/N)	65.0% (1118/1721)	62.9% (1303/2072)	0.197
Smoking history, % (n/N)	56.2% (968/1721)	14.9% (308/2072)	<0.001
Alcohol consumption history, % (n/N)	26.8% (461/1721)	1.2% (24/2072)	<0.001
Sedentary lifestyle, % (n/N)	43.9% (755/1721)	46.4% (961/2072)	0.130
Hypertension, % (n/N)	75.7% (1302/1721)	81.0% (1679/2072)	<0.001
Diabetes duration			<0.001
<1 year, % (n/N)	7.2% (123/1717)	5.8% (119/2067)	
1–5 years, % (n/N)	27.5% (472/1717)	22.6% (468/2067)	
5–10 years, % (n/N)	22.8% (391/1717)	22.9% (473/2067)	
≥10 years, % (n/N)	42.5% (731/1717)	48.7% (1007/2067)	
HbA1c (%), mean ± SD (n)	7.47 ± 1.751 (1719)	7.50 ± 1.727 (2069)	0.597
SBP (mmHg), mean ± SD (n)	135.3 ± 16.12 (1721)	136.9 ± 16.79 (2072)	0.003
DBP (mmHg), mean ± SD (n)	79.1 ± 9.54 (1721)	78.0 ± 9.81 (2072)	0.001
LDL-C (mmol/L), mean ± SD (n)	2.54 ± 0.898 (1718)	2.82 ± 0.935 (2068)	<0.001
TC (mmol/L), mean ± SD (n)	4.46 ± 1.309 (1721)	4.98 ± 1.286 (2072)	<0.001
TG (mmol/L), mean ± SD (n)	1.79 ± 1.534 (1717)	2.02 ± 1.563 (2067)	<0.001
HDL-C (mmol/L), mean ± SD (n)	1.18 ± 0.458 (1720)	1.31 ± 0.494 (2072)	<0.001

SD, standard deviation; BMI, body mass index; HbA1c, glycated hemoglobin; SBP, systolic blood pressure; DBP, diastolic blood pressure; LDL-C, low-density lipoprotein cholesterol; TC, total cholesterol; TG, triglyceride; HDL-C, high-density lipoprotein cholesterol.

### Pharmacotherapies

As shown in Tables 2, 48.2% of men and 38.1% of women were receiving treatment with lipid-lowering agents. Among these patients, the proportions receiving a combination therapy were quite low; more than 98.8% of patients received monotherapy, with no significant difference between men (98.8%) and women (98.9%). The most frequently reported lipid-lowering agents were statins (94.3%), followed by fibrates (3.7%) in all patients. Xue Zhi Kang (a traditional Chinese medicine for lowering lipid levels) was more widely prescribed in women than men (p < 0.001). The percentages of patients taking a lipid lowering agent who used statins (94.2% vs. 94.4%; p = 0.915), fibrates (4.3% vs 3.0%; p = 0.189), and nicotinic acid (0.8% vs. 0.4%; p = 0.344) were similar in men and women. The combinations of anti-diabetic and anti-hypertensive agents are listed in Supplemental Table 2.

**Table 2:** Comparison of lipid-lowering drugs prescribed in men and women.

	Men (N = 1721)	Women (N = 2072)
Treatment with lipid-lowering agents	48.2% (829/1721)	38.1% (790/2072)
Monotherapy	98.8% (819/829)	98.9% (781/790)
Dual therapy	1.2% (10/829)	1.1% (9/790)
Type of lipid-lowering agent Statins	94.2% (781/829)	94.4% (746/790)
Fibrates	4.3% (36/829)	3.0% (24/790)
Nicotinic acid	0.8% (7/829)	0.4% (3/790)
Xue Zhi Kang*	0.7% (6/829)	2.3% (18/790)
Others	1.0% (9/829)	0.9% (8/790)

Data are shown as % (n/N).

*Xuezhikang is an extract of cholestin from red yeast rice (Monascus purpureus) that each capsule contains 2.5 to 3.2 mg monacolin K (lovastatin), unsaturated fatty acids, essential amino acids, and small quantities of lovastatin hydroxyl acid, ergosterol and other components. Xuezhikang is categorized within the statin class by the lipid guidelines, primarily due to lovastatin as its main ingredient but it is in the TCM category.

### Relative differences in LDL-C goal attainment rates between men and women

**Figure 1:**
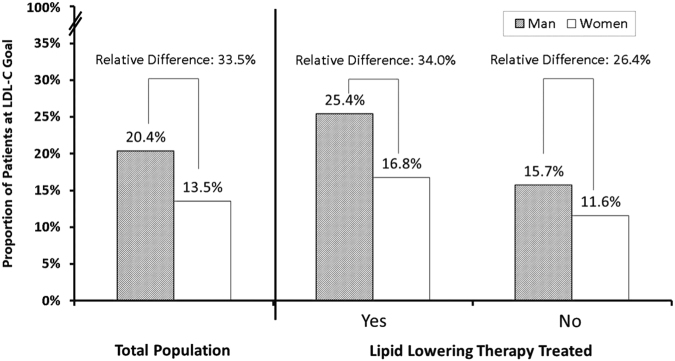
Relative differences in LDL-C goal attainment.

The LDL-C levels in women were significantly higher than those in men, regardless of lipid lowering therapies (Supplemental Table 3). As shown in Fig. 1, a lower percentage of women achieved LDL-C goal attainment compared with men (13.5% vs. 20.4%, p < 0.001), with a 33.5% relative difference in LDL-C goal attainment rates of women and men. The relative difference in LDL-C goal attainment was greater among patients aged ≥60 years (35.2%), patients with a history of cigarette smoking (38.5%), patients with hypertension (38.5%), and patients with a longer duration of diabetes (34.4%). However, the disparity was not observed between women and men with a history of alcohol consumption. The exact LDL-C goal attainment for women and men and the relative differences according to the characteristics of the study population are summarized in Table 3.

**Table 3:** Relative differences in LDL-C goal attainment between men and women.

		Men	Women	Relative difference*
All patients		20.4% (350/1718)	13.5% (280/2068)	33.5%
Age	≥60 years	20.8% (258/1239)	13.5% (230/1705)	35.2%
	<60 years	19.4% (92/475)	13.4% (48/359)	31.0%
Smoking history	Yes	20.6% (199/967)	12.7% (39/308)	38.5%
	No	20.1% (151/751)	13.7% (241/1760)	31.9%
Alcohol consumption history	Yes	19.6% (90/460)	20.8% (5/24)	−6.5%
	No	20.7% (260/1258)	13.5% (275/2044)	34.9%
Sedentary lifestyle	Yes	21.5% (162/753)	14.5% (139/959)	32.6%
	No	19.5% (188/965)	12.7% (141/1109)	34.7%
Diabetes duration	≥5 years	21.2% (237/1120)	13.9% (205/1477)	34.4%
	<5 years	18.9% (112/594)	12.5% (73/586)	33.9%
Lipid-lowering therapy	Yes	25.4% (210/827)	16.8% (132/788)	34.0%
	No	15.7% (140/891)	11.6% (148/1280)	26.4%
Hypertension	Yes	21.5% (279/1300)	13.2% (221/1675)	38.5%
	No	17.0% (71/418)	15.0% (59/393)	11.6%

LDL-C, low-density lipoprotein cholesterol.

*Relative differences (%) were calculated as (attainment in men − attainment in women)/attainment in men × 100%.

### Risk factors for not attained LDL-C goal by gender

Logistic regression analyses were performed to examine the association between gender and LDL-C goal attainment in diabetic outpatients with CHD. These analyses produced an unadjusted OR of 0.61 (95% CI: 0.52–0.73) for women compared with men. Furthermore, multivariate logistic regression analyses for both men and women revealed that only use of LLT was significantly associated with LDL-C goal attainment after adjustment for the factors of age, smoking history, alcohol consumption history, sedentary lifestyle, BMI, diabetes duration, and hypertension (Table 4).

**Table 4:** Risk factors for not attained LDL-C goal in the multivariate analysis.

	OR	Men	P	OR	Women	p
		95% CI			95% CI	
Age (≥60 vs. <60 years)	1.05	0.80–1.38	0.729	0.97	0.69–1.36	0.852
Smoking history (Yes vs. No)	0.89	0.66–1.21	0.463	1.20	0.88–1.64	0.254
Alcohol consumption history (Yes vs. No)	1.31	0.88–1.94	0.181	0.99	0.65–1.50	0.954
Sedentary lifestyle (Yes vs. No)	1.12	0.88–1.42	0.348	1.13	0.87–1.46	0.347
BMI (≥24 vs. <24 kg/m^2^)	0.92	0.72–1.19	0.526	1.22	0.93–1.61	0.147
Diabetes duration (≥5 vs. <5 years)	1.16	0.90–1.50	0.251	1.14	0.85–1.52	0.367
Receiving lipid-lowering agents (Yes vs. No)	1.82	1.42–2.32	<0.001	1.52	1.17–1.97	0.001
Hypertension (Yes vs. No)	1.30	0.97–1.74	0.081	0.82	0.59–1.12	0.208

LDL-C, low-density lipoprotein cholesterol; BMI, body mass index; OR, odds ratio; CI, confidence interval.

## Discussion

To the best of our knowledge, this is the first study to investigate the gender disparities in lipid level goal attainment among Chinese type 2 diabetes patients with concomitant CHD. In this study, we performed an analysis in a nationally representative sample of the diabetic population in China, and the main findings of our analysis were as follows: (1) the mean levels of LDL-C, TC, and TG in women with type 2 diabetes were higher than those in men; (2) fewer women received LLT compared with men; (3) irrespective of age, women were less likely to attain their LDL-C goal; and 4) only LLT was independently associated with LDL-C goal attainment in this population of diabetes patients with concomitant CHD. Our study provides a nationally representative example of the gender imbalance in the effectiveness of LLT and the achievement of lipid control in diabetes patients with CHD in China. These finding highlight the importance of initiatives to develop a tailored lipid management strategy for women that is able to overcome these imbalances and support optimal cardiovascular outcomes.

In the current study, female patients with type 2 diabetes and CHD had a higher mean LDL-C level, but received less LLT compared with their male counterparts. Female patients with type 2 diabetes and CHD were less likely to be treated with lipid-lowering agents than males (38.1% vs. 48.2%, with a disparity of 10.1% in the initiation of LLT between the men and women. Our findings are consistent with those of previous studies in that women with diabetes tended to have a higher LDL-C level than diabetic men^18,19^. The overall percentages of patients who reached their LDL-C treatment goal were 13.5% in women and 20.4% in men, representing a 33.5% relative difference in LDL-C treatment goal attainment between the men and the women. This finding is consistent with the latest published EUROASPORE project^20^, which demonstrated that men are more likely to be sufficient lipid control than women regardless of age and education. Notably, in our study, lower LDL-C goal attainment rates were consistently observed in female diabetes patients independent of the use of lipid-lowering medications. These findings revealed that gender differences in lipid management existed with respect to both initiation of LLT and LDL-C treatment goal attainment. A recently published study^21^ involving 9,950 patients with CHD indicated that female patients were prescribed insufficient doses of statins or combination LLT and were less likely to achieve their optimal LDL-C goals. Another national survey in the US^22^ including 2708 patients with CHD who received LLT indicated that goal attainment in women was 25% less than that in the men. Lack of LDL-C goal attainment is a global concern that also has been reported to affect the general population of France^23^ and Japanese diabetes patients with a history of CHD^24^. Our finding is consistent with the latest epidemiologic evidence, that only 42% of the 33000 high cardiovascular risk patients in Japan treated with statin^25^. Together, these findings highlight the need for more intensive treatment in dyslipidemia, and the importance of overcoming the lipid lowering treatment disparities.

Many factors likely contribute to the low rates of LDL-C treatment goal attainment in real-world clinical settings. In the present study, we aimed to identify possible risk factors for the lower LDL-C treatment goal attainment in type 2 diabetes patients. The multivariable regression analyses indicated that the OR for lipid attainment due to gender difference was 0.61 for women compared with men after adjusting for age, diabetes duration, BMI, hypertension, use of LLT, smoking history, and alcohol consumption history. Our findings are similar to those of an observational study in the US^26^, in which women with diabetes were 0.70 times (95% CI 0.58–0.86) less likely to attain LDL-C goals than male patients. The underlying mechanisms responsible for poorer lipid control among women with diabetes remain unclear. One possible explanation for the gender disparity in LDL-C treatment goal attainment may be lower adherence to medication in women^27,28^. A recent published survey shows that in a total of 10,138 adults’ survey about understanding statin use in America, women were more likely to stop or switch their statin than men because of new or worsening muscular symptoms^29^. The risk of statin-related muscular symptoms may be higher in patients with certain concomitant medical conditions such as diabetes^30^. AHA scientific statement also mentioned that myalgia may be more frequent in women. Apart from adherence to LLT, the intensity of lipid-lowering medication regimens may also be related to LDL-C goal achievement, with lower intensity regimens being less likely to support goal attainment^31^. However, adverse effects from intensified regimens may increase non-adherence. The lower rates of LDL-C goal attainment in diabetic women not on LLT may be associated with the higher basal lipid profiles in our study. Thus, gender differences in baseline lipid levels offer another explanation^32^. It remains unclear whether the underlying causes of the observed disparities are mainly biological or behavioral.

Our results have significant implications for clinical practice. Physicians should attempt to narrow the gender disparities in lipid-lowering goal attainment by increasing the amount of attention currently devoted to female patients. The ADA recommends the addition of statins to lifestyle modification irrespective of baseline lipid levels in diabetes patients with a history of cardiovascular disease^33^. We found that women were much younger and fewer were current smokers, which are the established risk factors for both all-cause and cardiovascular death; thus, it is not surprising that mortality is lower in IGT women. However, the reduction in mortality mainly occurred in women (HR = 0.46, p < 0.05) and the lifestyle intervention seemed to have little effect in men (HR = 0.97, p > 0.05). Moreover, female diabetes patients should receive more adequate treatment to support lipid-lowering goal attainment for LDL-C^34^. Finally, acknowledging gender disparities in lipid goal attainment and CHD risk in clinical guidelines may help to close the gap between men and women in terms of achieving the LDL-C goal and hopefully reducing the gender disparities in the risk of CHD. More epidemiologic studies are needed to set the gender specified threshold or cut-off points for lipid management in diabetes patients with established CHD.

The present study has several limitations. First, our study was a cross-sectional observational study and causal relationships could not be established because the presence of risk factors and LDL-C goal attainment were measured simultaneously. Moreover, the disparities in baseline serum lipids profile may partly contribute to the differences in long-term lipid control, and this study could not determine the exact reasons for worse lipid control among women with diabetes. The collection of information about risk factors was also retrospective, creating a risk of recall bias. Well-designed cohort studies are needed to prospectively investigate the gender disparities. Second, our patient population represents only those receiving outpatient treatments in China, and thus, may not be representative of severe cases. Third, apart from gender, the baseline LDL-C level and dosage of statin treatment also contributed to the gender disparities in LDL-C-lowering goal attainment. However, we were unable to investigate the intensity of the lipid-lowering medication regimen and medication adherence in our analysis model. Fourth, CHD is a very difficult clinical condition and the diagnosis methods and judgment vary considerably. Therefore, bias exists and the atherosclerotic degree or the severity of coronary lesions among our population was different in this real-life study. More well-designed studies are needed to prospectively verify our findings. Finally, lipid parameters were not measured in a central core laboratory to ensure the accuracy of the measurements.

In conclusion, this study demonstrates that among outpatients with type 2 diabetes and concomitant CHD, the lipid goal attainment rate is lower in women than in men. Recognition of the gender disparities in lipid-lowering goal attainment among diabetes patients can support the development of individualized management for women with diabetes. More well-designed longitudinal studies are needed to determine the causes of the identified gender disparities and to tailor interventions accordingly.

## Methods

### Study population

This study was a post hoc analysis of the CCMR-3B. The CCMR-3B was an observational, cross-sectional, multicenter, multispecialty study conducted to investigate blood glucose, blood lipid, and blood pressure control status at 104 hospitals across China from August 2010 to March 2011 (registered in clinicaltrials.gov, NCT01128205). A total of 25,454 adults with type 2 diabetes treated as outpatients at 104 hospitals were eligible for inclusion in the study. The details of population sampling have been described previously^35^. This research protocol was approved by the Ethics Committee of People’s Hospital, Peking University, and the study was conducted in accordance with the Good Clinical Practice and the International Conference on Harmonization guidelines. All participants provided written informed consent at the initial enrollment.

Patients were eligible if they: (1) were aged 18 years or older, had been diagnosed with type 2 diabetes in accordance with the American Diabetes Association criteria^36^ and China Guideline for Type 2 Diabetes^37^ and had been diagnosed with concomitant CHD; (2) had diabetes for a duration of at least 6 months before enrollment; and (3) had a documented fasting lipid profile during the previous 6 months and after lipid-lowering therapy (LLT) for at least 3 months. Established CHD was defined as patients with clear medical record of CHD diagnosis or patients newly diagnosed as CHD by cardiologists according to clinical guidelines on the present study visit. Namely, CHD is defined by the presence of stenosis ≥70% of the diameter of at least one segment of a major epicardial artery, or stenosis ≥50% of the diameter of the left main disease during invasive coronary angiography. Patients with type 1 diabetes and gestational diabetes were excluded from this study.

### Data collection

During the single outpatient visit, all participants completed a self-administered standardized data collection form at enrollment. The following data were obtained from the questionnaires: age at time of enrollment, gender, educational level, marital status, occupational status, income, physical activities, smoking and alcohol consumption history, individual and family medical history, previous diagnosis of dyslipidemia or hypertension, and use of antihypertensive or lipid-lowering agents. Body weight, height and waist circumference were measured using standard methods. Blood pressure was measured two times in a seated or supine position with a mercury column sphygmomanometer after at least 5 minutes of rest before the initial blood pressure reading was recorded. Blood pressure was the average of the first and second measurements recorded more than 2-minutes interval. Well-documented fasting serum glucose, fasting lipid profile including total cholesterol (TC), LDL-C, HDL-C, and triglycerides (TG) measurements obtained within 1 month were recorded. A hemogloblin A1c (HbA1c) concentration known to have been obtained during the 3 months prior to the enrollment visit, or obtained at enrollment, was recorded.

### Definition of variables

Diabetes was defined by self-reporting of a prior history of diabetes and confirmed by a fasting plasma glucose level ≥7.1 mmol/L and/or data on current insulin or hypoglycemic medication use regardless of the fasting plasma glucose level. Hypertension was defined as an average BP ≥140/90 mmHg or a previous history of hypertension. Body mass index (BMI) was calculated as body weight in kilograms divided by height in meters squared (kg/m^2^). The lipid-lowering goal attainment was defined as the percentage of patients achieving their LDL-C treatment goal (LDL-C <1.8 mmol/L) with lipid-lowering agents according to the recommendation of the National Cholesterol Education Program Expert Panel on Detection, Evaluation, and Treatment of High Blood Cholesterol in Adults^38^. Waist circumference was measured at the minimal horizontal girth between the rib cage and the iliac crest. Sedentary behavior was evaluated according to the amount of time spent watching television or using a computer and sitting time at place of work or home. A history of smoking was defined as having smoked on average one cigarette per day for at least 1 year. A history of alcohol consumption was defined as having consumed on average 50 g alcohol per day for 1 year or longer.

### Statistical analysis

Continuous variables with normal distribution are expressed as mean ± standard deviation (SD) or median with interquartile range (non-normal distribution). Comparisons between groups were examined using one-way analysis of variance (ANOVA) test or Mann-Whitney U test for continuous variables. The Pearson chi-squared test or Fisher exact test was used for analysis of categorical data. The relative differences (%) in blood lipid (using LDL-C measurement) attainment rates between men and women were calculated as (attainment rate in men - attainment rate in women)/attainment rate in men × 100%. The Cochran-Mantel-Haensel (CMH) test was used to calculate the relative differences (%) in lipid-lowering goal attainment rates between men and women according to the characteristics of the study population. A multivariate logistic regression analysis with LDL-C goal attainment as a dependent variable was applied to identify the potential risk factors. First, we used a basic model to examine the independent impact of gender on LDL-C goal attainment. The results are expressed as odds ratios (ORs) and 95% confidence intervals (CIs). Then each potential factor including age, smoking history, alcohol consumption history, sedentary lifestyle, BMI, diabetes duration, hypertension, or use of lipid-lowering agents was added to the base model to calculate the percent change in the OR for gender after adding each variable. The percent change in the OR after the addition of a specified variable to the model was calculated using the following formula: [(OR1 − OR2)/(OR1 − 1.0)] × 100%, where OR1 represents the OR for gender derived from the base model, and OR2 represents the OR for gender after the addition of the designated variable. All the statistical analyses were performed with SAS version 9.2 (SAS Institute Inc., Cary, NC, USA). A statistically significant difference was considered at the two-tailed level of p < 0.05.

## Electronic supplementary material

Supplementary Information
