# Supplementary material for: Gender Disparities in Lipid Goal Attainment among Type 2 Diabetes Outpatients with Coronary Heart Disease: Results from the CCMR-3B Study
Source: Sci Rep. 2017 Oct 4;7:12648. doi: 10.1038/s41598-017-13066-z (PMC5627285; doi:10.1038/s41598-017-13066-z)
Supplement: Supplementary file 1 — Supplementary Information [file 41598_2017_13066_MOESM1_ESM.pdf]

Gender Disparities in Lipid Goal Attainment among Type 2 Diabetes Outpatients with Coronary Heart Disease: Results from the CCMR-3B Study

Xiaomei Zhang[1], Linong Ji[2,*], Xingwu Ran[3], Benli Su[4], Qiuhe Ji[5], and Dayi Hu[6]

[1]Peking University International Hospital, Department of Endocrinology and Metabolism, Beijing, 102206, China
[2]Peking University People's Hospital, Department of Endocrinology and Metabolism, Beijing, 100044, China
[3]West China Hospital, Sichuan University, Department of Endocrinology and Metabolism, Chengdu, 610041, China [4]The Second Affiliated Hospital of Dalian Medical University, Department of Endocrinology, Dalian, 116027, China [5]Xijing Hospital, Forth Military Medical University, Department of Endocrinology, Xi'an, 710032, China
[6]Peking University People's Hospital, Department of Cardiology, Beijing, 100044, China
*jiln@bjmu.edu.cn

Supplementary information

1. Supplementary Table 1: Patients distribution and social-economic status
2. Supplementary Table 2: Anti-diabetes and Anti-hypertension treatment in patients
3. Supplementary Table 3: Lipid profile in Lipid lowering treated and not treated patients.
4. Appendix 1: Investigators List

Supplementary Table 1. Patients distribution and social-economic status

	Men (N=1721)	Women (N=2072)
Regions		
North	17.1% (295)	17.7% (366)
Northeast	15.9% (273)	16.5% (341)
East	22.1% (380)	15.6% (324)
South	9.6% (165)	15.3% (318)
Northwest	14.8% (255)	16.1% (333)
Southwest	20.5% (353)	18.8% (390)
Hospital Tiers		
Tier 1	26.8% (462)	26.3% (544)
Tier 2	34.6% (595)	36.9% (765)
Tier 3	38.6% (664)	36.8% (763)
Department		
Cardiology	14.5% (250)	12.1% (251)
Endocrinology	46.0% (791)	47.9% (992)
Nephrology	6.2% (107)	6.4% (133)
Internal Medicine	33.3% (573)	33.6% (696)
Residence		
Urban	95.1% (1637)	93.1% (1929)
rural	4.9% (84)	6.9% (143)
Education		
Illiterate	2.5% (43)	11.3% (234)
Primary School	16.3% (281)	29.4% (609)
Middle School	48.5% (835)	44.2% (916)
College	18.9% (325)	10.8% (224)
Postgraduate	13.8% (237)	4.3% (89)
Employment		
Unemployed	4.8% (82)	13.3% (276)
Full-time Job	14.3% (246)	2.2% (46)
Part-time Job	2.6% (44)	0.7% (14)
Retired	78.4% (1349)	83.8% (1736)
Medical Insurance covered		
No	4.5% (78)	4.7% (98)
Yes	95.5% (1643)	95.3% (1974)
Home Revenue (RMB per month)		
<2000	30.3% (521)	43.6% (903)
2000-5000	52.4% (902)	45.1% (934)
>5000	14.2% (244)	7.4% (153)
Unknown	3.1% (54)	4.0% (82)

Data are shown as % (n).

Supplementary Table 2. Anti-diabetes and Anti-hypertension treatment in patients

	Men	Women
Anti-diabetes Agents		
Metformin	42.1% (725/1721)	35.0% (725/2072)
Sulfonylureas	23.4% (403/1721))	30.3% (628/2072)
α-glucosidase inhibitor	28.6% (492/1721)	27.0% (559/2072)
Thiazolidinedione	3.0% (52/1721)	8.1% (167/2072)
Meglitinides	7.3% (126/1721)	4.4% (91/2072)
Others	1.0% (17/1721)	1.2% (24/2072)
Anti-hypertensive drugs in hypertension patients		
Diuretics	3.0% (39/1302)	4.2% (71/1679)
β-blocker	16.2% (211/1302)	8.2% (138/1679)
Calcium Channel Blockers	38.4% (500/1302)	41.3% (693/1679)
ACE Inhibitor	11.8% (154/1302)	15.2% (255/1679)
Angiotensin II Receptor Antagonist	22.8% (297/1302)	30.5% (512/1679)
α-blockers	2.7% (35/1302)	8.1% (136/1679)

Data are shown as % (n/N).

S3 Table. Lipid profile in Lipid lowering treated and not treated patients.

	Treated			Not Treated		
	Women	Man	*p*	Women	Men	*p*
TC	4.89±1.29	4.32±1.40	<0.001	5.04±1.28	4.58±1.21	<0.001
LDL-C	2.77±0.99	2.45±0.93	<0.001	2.84±0.90	2.62±0.86	<0.001
HDL-C	1.25±0.41	1.11±0.38	<0.001	1.35±0.59	1.23±0.51	<0.001
TG	2.05±1.54	1.84±1.52	0.005	1.99±1.57	1.72±1.54	<0.001

Appendix 1

Investigators List (by hospital)

1. Peking University People's Hospital
   Zhun Sui, Mei Wang, Yingli Chen, Rongjing Ding, Dayi Hu, Linong Ji

2. Beijing Hospital, Ministry of Health
   Lixin Guo, Qi Pan, Hua Wu

3. Xuanwu Hospital Capital Medical University
   Huili Chen, Haixia Hou, Qi Hua, Yanling Wang

4. Anzhen Hospital Capital Medical University
   Hong Chen, Xin Du, Changsheng Ma, Hong Tao

5. Peking University First Hospital
   Yong Huo

6. Peking Union Medical College Hospital
   Yuxiu Li, Zengyi Li, Xiaoping Xing

7. China-Japan Friendship Hospital
   Yanyan Chen, Guangwei Li, Wenge Li, Xiaoxia Shen

8. The General Hospital of the People's Liberation Army
   Nan Jin, Jing Li, Yiming Mu, Changyu Pan

9. Beijing Shi Jing Shan Hospital
   Guogang Li, Weihua Li, Shanshan Lin, Changyang Su, Mingsheng Wang

10. China Meitan General Hospital
    Hongmei Li, Zhenwei Shi

11. Huguoshi Chinese Medicine Hospital Beijing University of Chinese Medicine
    Jian Huang, Bei Yang

12. Daxing Chinese Medicine Hospital Beijing University of Chinese Medicine
    Li Ma, Jie Zhang

13. Beijing Pinggu Hospital
    Yufeng Li, Cuiling Zhao

14. Nanyuan Hospital
    Kaijie Yang, Jixia Zhao

15. Shichahai Community Health Service of Xichen District Beijing
    Junqing Liu

16. Beijing University of Posts and Telecommunications Community Health Service Center
    Xiaoyan Wang

17. Puhuangyu Community Health Service Center of Beijing
    Yan Zhang

18. Beijing Jiaotong University Hospital
    Manhong Li

19. The Second Affiliated Hospital of Dalian Medical University
    Zhuo Li, Benli Su, Jiuyang Zhao, Lin Zhao

20. Shenyang No. 4 People's Hospital
    Yanhe Cui, Jinsong Kuang, Man Li, Yinjun Li, Weiguang Luo

21. Affiliated Zhongshan Hospital of Dalian University
    Shaokui Liu, Xiaomei Wang

22. Second Affiliated Hospital of Jilin University
    Bin Liu, Yu Liu, Lijuan Wang

23. Liaoning Power Center Hospital

Mingyu Gao

24. The Fourth Affiliated Hospital of Chinese Medicine University

Zhiying Duan, Hongmei Ji, Yuanzhe Jin, Zongqian Wang

25. Dalian Lvshunou People's Hospital

Lu Chen, Danqi Wu, Shumin Yang

26. Dalian Fifth People's Hospital

Xingjia Liu, Mingrui Lv, Yili Ma, Li Wang, Rui Yu

27. Diabetes Treatment Center Of Liaoning Province

Bin Gao

28. China–Japan Union Hospital and The First Affiliated Hospital of JILIN University

Feng Liu, Qing Wang, Dongyan Xu, Ping Yang

29. Changchun People's Hospital

Pin Fu, Kaiping Mu

30. Changchun Chaoyang District People's Hospital

Ruijie Meng, Yu Zhao

31. Dalian ShaHeKou XingHaiWan Garden Community Health Service Center

Tinggui Gao, Yunshun Li

32. Dalian ShaHeKou HeiShiJiao baiyun Community Health Service Center

Shuqin Bao

33. Shenyang HuangGuOu Longjiang Community Health Service Center

Xia Cao

34. Tiexiqu WeiGong Community Health Service Center

Xianying Liu, Jianbo Zhao, Shenyang

35. Xijing Hospital

Qiuhe Ji

36. Chinese PLA 323 Hospital

Peijun Mao, Jixian Ye

37. Chinese PLA 451 Hospital

Dafang Wu, Yan Zhou

38. The Second Hospital of Lanzhou University

Jiangong Ren

39. Gansu Provincial Hospital

Jin Liu

40. Xinjiao People’s Hospital of Haizhu District Guangzhou

Zhaoqi He, Zhen Que

41. Red Cross Hospital of Haizhu District Guangzhou

Liping Li, Zhizhao Zhou

42. Hulin Street Community Health Service Center of Liwan District Guangzhou

Yi Lin, Xiangmin Xu

43. Hongshan Street Community Health Service Center of Kaifu District Changsha

Cuiwei Zheng

44. West China School of Medicine Sichuan University

Dawei Chen, Ping Fu, Xing Kang, Fang Liu, Xingwu Ran

45. Second people's hospital of Chengdu

Xiaoyun Chen, Bing Huang, Qiu Li, Xiance Luo

46. First People's Hospital Of Chengdu

Jie Gao, Zheng Li, Zhiming Lu, Yahui Yan

47. Xinqiao Hospital of Third Military Medical University

Zihui Xu, Houdi Zhou

48. The First affiliated Hospital of Chongqing Medical University

Han Lei, Qifu Li, Changhong Zhao

49. The Second affiliated Hospital of Chongqing Medical University

Yan Cheng, Changhui Guo, Shiguo Tang, Yanping Xu, Gangyi Yang,Yuehui Yang

50. Hospital of Tradition Medicine LS.SC

Kang Peng, Fenyuan Wu

51. Hospital of Tradition Medicine MS.SC

YangHong Wang

52. Sixth People's Hospital Of Chengdu

Yu Wang

53. Fudan University Huashan Hospital

Jing Chen, Chuanming Hao, Rengming Hu, Yun Li, WeiLing Qi, Haiming Shi, Chaoyun Zhang

54. The First Affiliated Hospital of Shanghai JiaoTong University

Yun Jiang, Shaowen Liu, Yongde Peng, Wei Wang, Weijie Yuan, Qing Yu

55. Huadong Hospital Affiliated of Fudan University

Lei Cai, Jiao Sun

56. Shanghai Changzheng Hospital

Ru Ding, Zhimin Liu, ChangLin Mei , Suxing Tuo, Zonggui Wu, Bei Zhang

57. Tongji University affiliated Tenth People's Hospital

Ai Peng, Shen Qu, Yaxiang Song, Yidong Wei, Yawei Xu, Peng Yang

58. Jiangsu province hospital

Tao Yang, Qingxin Yuan

59. Shanghai Post and Telecommunications Hospital

Yi Xin

60. Shanghai Pudong New Area public Hospital

Mingjun Gu, Meihua Guo, Lianyong Liu, Jun Lu, Jianping Qiu, XiaoLing Pi, Xumin Shen

61. Shanghai Central Hospital of Shanghai Changning District

Shan Huang, Xiaohong Jiang

62. Central Hospital of Shanghai Minxing District

Chengjun Chen, Xia Chen, Xudong Xu, Jialin Yang, Dadong Zhang, Yu Zhang

63. Shanghai Luwan District Central Hospital

Ling Chen, Yuwei Huang, Hui Li, Qiang Lu, Hong Shao, Ying Shen, Jianrong Zhao, Zhen Zhu

64. Central Hospital of Shanghai Jingan District

Jianrao Lu, Jun Wang, Xiu'e Zhao

65. Central Hospital of Shanghai Songjiang District

Xiujuan Zang, Lijuan Zhang

66. Shanghai Yangpu District Yin hang Community Health Service Center

Rong Li, Yumei Ma

67. Shanghai Yangpu District Bridge Community Health Service Center

Ming Cui, Yanhong Zhou

68. Shanghai Jingan District community health service center in Jiangning

Weifeng Chen,Tianhan Shen

69. Shanghai Qingpu District Zhao Zhen Community Health Service Center

Qing Gu, Meijuan Zhu

70. Shanghai Minhang District Urban Community Health Service Center

    Hua Hang, Chunxiao He

71. The Second People's Hospital Haizhu District Guangzhou

    Yonglian Xie, Jixuan Zhao, Minling Zhao

72. The Second People's Hospital of Shanxi

    Wenli Liu, Lili Pa

73. Liwan Hospital Guangzhou Medical University

    Hongbing Luo

74. Workers' Hospital of Xi'an Aero-engine Group Co., Ltd.

    Yu'nan Jia

75. Xi An Huxian Hospital

    Yun'an Wang

76. Zhongshanmen Community Medical Service Center of Xinchen District of Xi'an

    Yipeng Hou, Bing Xia

77. The Second Affiliated Hospital of Sun Yet-sun Hospital

    Guojuan Lao, Yan Li

78. Xi An Hansengzhai Community Medical Service Center of Xinchen District

    Jiang Wu,

79. Shangde Hospital of Xi'an

    Mingzhao Yang

80. The Third Affiliated Hospital of Sun Yet-sun Hospital

    Ling Chen, Hongrong Deng, Ruimin Dong, Jin Li, Xun Liu, Tanqi Lou, Jianping Weng, Longyi Zeng

81. Xi An Hujiamiao Hospital of Xinchen District

Ji Li

82. The First Affiliated Hospital of Sun Yet-sun Hospital

Ailing Chen, Yanbing Li

83. General Hospital of Guangzhou Military Command of PLA

Aiming Deng, Jian Qiu, Junrong Tong, Lin Xu, Lili Ye, Xianyang Zhong

84. Nanfang Hospital

Ying Cao, Xiaojing Hu, Yaoming Xue

85. Xiangya Hospital Central-South University

Meng Jiang, Mingxiang Lei

86. The Second Xangya Hospital Central-South University

Weili Tang, Yu Zhou, Zhiguang Zhou

87. Bingong 521 Hospital Xi'an

Xudan Zhen

88. Dongfeng Branch of the Second People's Hospital Yuexiu District Guangzhou

Xiangwei Chen

89. Guangzhou Diabetes Hospital

Maolin Luo

90. Finance & Trade Hospital of Hunan Province

Xiao Xiong, Liping Zhu

91. Xi An Huashan Centre Hospital

Qinli Fan

92. First People's Hospital Of Longquanyi District Of Chengdu

Lihua Liu, Li Zhong

93. People's Hospital of Pi County

Tianhu Liu, Yumei Zhang

94. Chongqing Tung Wah Hospital

Jinghui Lu

95. Wuhou Community Health Service Center of Wuhou District of Chengdu

Lin Yang, Wenyi Yang

96. West City Community Health Service Center of Xindu District of Chengdu

Liuli He, Xiaohua Wu

97. Yuling Community Health Service Center of Wuhou District of Chengdu

Xilian Gao, Jing Jiang

98. Southwest Computer Company Worker's Hospital of Chongqing

Yana Xu, Bo Zhang

99. Yuxi Hospital of Chongqing

Rongping Tian, Xinjian Zhou

100. General Hospital Of Chongqing Iron And Steel

Xiaochun Teng

101. Sichuan Academy of Medical Sciences & Sichuan Provincial People’s Hospital

Mingjing Bao, Pengqiu Li, Limei Li, Yichuan Wu, Yang Xuan, Yan Yang, Xuejun Zhang

102. Third people's hospital Chengdu

Hui He, Jingyu Liu, Li Zhang

103. Second People's Hospital Of Jiulongpo District Of Chongqing

Qiu Xu, Hongju Zhong
